# Perineural invasion and the “cold” tumor microenvironment in pancreatic cancer: mechanisms of crosstalk and therapeutic opportunities

**DOI:** 10.3389/fimmu.2025.1650117

**Published:** 2025-08-20

**Authors:** Jianbiao Xu, Hong Yao, Junfeng Wang, Yun Jin, Wei Chang, Lanjiang Li, Lei Zou

**Affiliations:** ^1^ Department of General Surgery, The First People’s Hospital of Yunnan Province, The Affiliated Hospital of Kunming University of Science and Technology, Kunming, Yunnan, China; ^2^ Department of Radiology, The First People’s Hospital of Yunnan Province, The Affiliated Hospital of Kunming University of Science and Technology, Kunming, Yunnan, China; ^3^ Department of Hepatobiliary and Pancreatic Surgery, The First People’s Hospital of Yunnan Province, The Affiliated Hospital of Kunming University of Science and Technology, Kunming, Yunnan, China; ^4^ Department of Epidemiology and Health Statistics, School of Public Health, Kunming Medical University, Kunming, Yunnan, China

**Keywords:** pancreatic ductal adenocarcinoma, perineural invasion, tumor microenvironment, immunosuppression, neuro-immune crosstalk, cancer-associated fibroblasts, tumor-associated macrophages, CXCL12/CXCR4

## Abstract

Pancreatic ductal adenocarcinoma (PDAC) remains a devastating malignancy characterized by profound lethality, aggressive local invasion, dismal prognosis, and significant resistance to existing therapies. Two critical biological features underpin the challenges in treating PDAC: extensive perineural invasion (PNI), the process by which cancer cells infiltrate and migrate along nerves, and a profoundly immunosuppressive, or “cold,” tumor microenvironment (TME). PNI is not only a primary route for local tumor dissemination and recurrence but also a major contributor to the severe pain often experienced by patients. Concurrently, the PDAC TME is typified by a dense desmoplastic stroma, hypoxia, and an abundance of immunosuppressive cells—including cancer-associated fibroblasts (CAFs), tumor-associated macrophages (TAMs), myeloid-derived suppressor cells (MDSCs), and regulatory T cells (Tregs)—while lacking sufficient infiltration of effector T cells, rendering it largely unresponsive to immunotherapies like checkpoint inhibitors. Although historically studied as separate entities, accumulating evidence reveals a deep-seated and complex bidirectional crosstalk between the neural components involved in PNI and the immune and stromal cells constituting the TME. Key cellular mediators, such as CAFs and TAMs, and shared signaling pathways, including the CXCL12/CXCR4 axis, TGF-β signaling, and neurotrophin pathways (e.g., NGF/TrkA), appear to act as critical nodes, coordinating the progression of PNI while simultaneously shaping and maintaining the immunosuppressive TME. This review synthesizes the current understanding of these intricate neuro-immune interactions in PDAC. We delineate the molecular and cellular mechanisms governing this crosstalk and explore how targeting these shared regulatory networks presents novel therapeutic opportunities, potentially disrupting PNI while concurrently “heating” the cold TME to overcome immunotherapy resistance. Elucidating this interplay is crucial not only for a deeper comprehension of PDAC’s invasive and metastatic mechanisms but also for uncovering new therapeutic vulnerabilities to improve patient outcomes.

## 1 Introduction

### 1.1 Pancreatic ductal adenocarcinoma: an unmet clinical challenge

Pancreatic ductal adenocarcinoma (PDAC) represents a major global health burden, characterized by a steadily increasing incidence and a mortality rate that closely mirrors its incidence (1, 2). It currently ranks as a leading cause of cancer-related deaths worldwide and is projected to become the second leading cause in Western countries by 2030 (3). The prognosis for PDAC patients remains exceptionally poor, with the overall 5-year survival rate being approximately 10-13%, a figure that has seen only marginal improvement despite decades of research (4).

PDAC typically arises from precursor lesions within the pancreatic ducts, such as pancreatic intraepithelial neoplasia (PanIN), or through acinar-to-ductal metaplasia (ADM) (5). Its development is driven by a characteristic sequence of genetic alterations, most notably activating mutations in the *KRAS* oncogene (present in >90% of cases) and inactivating mutations in tumor suppressor genes like *CDKN2A* (p16), *TP53*, and *SMAD4* (6). Established risk factors include smoking, chronic pancreatitis, obesity, family history, and notably, type 2 diabetes (7).

The clinical management of PDAC is challenging. Due to the lack of specific early symptoms and reliable screening methods, the majority of patients (~80%) are diagnosed at advanced stages, precluding potentially curative surgical resection (3). Even for the minority who undergo surgery, recurrence rates are exceedingly high (8). Furthermore, PDAC exhibits significant intrinsic and acquired resistance to conventional treatments, including chemotherapy and radiotherapy (9). Contemporary approaches, such as targeted therapies and immunotherapies, have yielded limited success in unselected patient populations (10, 11).

### 1.2 Perineural invasion and the immunosuppressive TME: key features of PDAC aggressiveness

Two biological characteristics are major contributors to the aggressive nature and resistance to therapy of PDAC: perineural invasion (PNI) and the unique tumor microenvironment (TME). PNI, the infiltration of cancer cells along and within nerve structures, is an almost universal histological hallmark of PDAC, observed in 70-100% of cases, often even in early precursor lesions (12). This neurotropic behavior is an active invasion pathway facilitating local tumor spread, contributing significantly to post-surgical recurrence, and generating the debilitating pain associated with the disease (13).

Parallel to PNI, the PDAC TME presents a formidable barrier to treatment. It is characterized by an extensive desmoplastic reaction—a dense fibrotic stroma rich in extracellular matrix (ECM) components that can constitute up to 90% of the tumor mass (14). This stroma creates a hypoxic, hypovascular, and high-pressure environment that impedes the delivery of therapeutic agents and the infiltration of immune cells. Crucially, the PDAC TME is profoundly immunosuppressive, often described as immunologically “cold” (15). It is heavily infiltrated by immunosuppressive cell populations, including various subtypes of cancer-associated fibroblasts (CAFs), M2-polarized tumor-associated macrophages (TAMs), myeloid-derived suppressor cells (MDSCs), and regulatory T cells (Tregs). Conversely, it typically lacks significant infiltration of cytotoxic CD8+ T lymphocytes (CTLs). This landscape is a primary reason for the failure of immune checkpoint inhibitors (ICIs) in most PDAC patients (15, 16).

### 1.3 The emerging significance of neuro-immune crosstalk in PDAC

Historically, research into PDAC progression often focused on PNI and TME immunology in relative isolation. However, a growing body of evidence indicates that these phenomena are closely interconnected through complex, bidirectional signaling pathways—a concept referred to as neuro-immune crosstalk (5). Nerves and their associated signaling molecules (neurotransmitters, neurotrophins) can directly influence the function of immune and stromal cells, contributing to the immunosuppressive milieu (17). Conversely, components of the TME, including cancer cells, CAFs, and immune cells like TAMs, secrete factors that actively promote nerve growth, remodeling, and invasion, thereby facilitating PNI (18, 19).

This review is centered on the hypothesis that this neuro-immune crosstalk is a fundamental aspect of PDAC biology, where shared mediators mechanistically link PNI and the establishment of the cold, immunosuppressive TME (20). We aim to synthesize the current understanding of these interactions, dissect the key mechanisms, and evaluate the potential of targeting this axis as a novel therapeutic strategy. By disrupting pathways that simultaneously drive nerve invasion and immune suppression, it may be possible to inhibit local spread, alleviate pain, and “heat up” the TME, rendering it more susceptible to immunotherapy and improving outcomes for patients with PDAC (21).

## 2 Perineural invasion in PDAC pathogenesis

### 2.1 Definition, prevalence, and pathological features

Perineural invasion (PNI) is defined histologically as the presence of cancer cells in close proximity to nerves, specifically within the epineural, perineural, or endoneural spaces of the nerve sheath (8). A commonly used criterion requires cancer cells to track along or surround at least 33% of the nerve’s circumference, or to be present within any of the three nerve sheath layers (8, 22). Some studies further distinguish between invasion confined to the perineurial space (PNI) and deeper invasion into the endoneurium, affecting Schwann cells and axons directly, termed endoneurial or intraneural invasion (ENI/INI) (23). This distinction may hold prognostic significance, as ENI has been associated with more severe pain and potentially worse outcomes compared to PNI alone (24).

While PNI occurs in various solid tumors, its prevalence in PDAC is exceptionally high, ranging from 70% to nearly 100% in surgical specimens, far surpassing rates seen in cancers of the prostate, head and neck, or colorectum (8). It is suggested that PNI could be detected in virtually all PDAC cases if sufficient pathological sections are examined (16). Importantly, PNI is not merely a feature of advanced disease; it is frequently observed in early-stage PDAC and even within precursor PanIN lesions, indicating it is an early event in pancreatic carcinogenesis (5). Pathologically, PNI in PDAC is often associated with “neural remodeling,” characterized by nerve hypertrophy (increased size), increased nerve density, neurogenic inflammation, and signs of neuronal damage (25).

### 2.2 Molecular and cellular mechanisms driving PNI

PNI is an active biological process involving reciprocal communication between cancer cells, neural cells (neurons and Schwann cells), and various components of the TME within a “perineural niche” (26). The process involves several key steps:

Mutual chemotaxis: Nerves and cancer cells attract each other. Cancer cells release neurotrophins like Nerve Growth Factor (NGF), Brain-Derived Neurotrophic Factor (BDNF), Glial Cell Line-Derived Neurotrophic Factor (GDNF), and artemin (ARTN), which bind to receptors on nerve cells, promoting neurite outgrowth toward the tumor (8). Conversely, neural structures release factors such as NGF, GDNF, ARTN, and CXCL12 (SDF-1), which act as chemoattractants for cancer cells expressing corresponding receptors (21). Pancreatic stellate cells (PSCs) contribute via tenascin C (27). This reciprocal signaling establishes a chemotactic gradient guiding cancer cell migration toward nerves.

Extracellular Matrix (ECM) remodeling: To invade, cancer cells secrete matrix metalloproteinases (MMPs), particularly MMP2 and MMP9, to degrade the surrounding ECM (8). The activation of these MMPs is driven by signaling pathways initiated by factors like GDNF (via RET-PI3K/AKT and RAS/ERK pathways), L1CAM (via STAT3), and galectin-1 (LGALS1) from PSCs (via SRC signaling) (8, 28). Macrophages, recruited by Schwann cell-derived CCL2, release Cathepsin B, which degrades collagen IV in the perineurium (29). CAFs are major contributors to this process by producing collagen and MMPs (30).

Adhesion and invasion: Cancer cells adhere to the nerve sheath. This process is mediated by specific adhesion molecules, such as Mucin 1 (MUC1) on cancer cells, binding to Myelin-Associated Glycoprotein (MAG) on nerves, and interactions involving NCAM1 and β1 integrin (8). Invasion is further promoted by factors released from damaged nerves (e.g., PAP/REG3A), signaling via SDC3/PTN, metabolic support (e.g., serine from axons), and factors from Schwann cells (e.g., TGF-β enhancing invasiveness, CCL2 recruiting macrophages) (20). Schwann cells can even create “tracks” (TASTs) that guide cancer cells as they migrate (31).

Immune evasion within the perineural niche: Cancer cells evade local immune surveillance, a process aided by neurotransmitters like acetylcholine (ACh) (5) and norepinephrine (NE) (8) that suppress CD8+ T cell function. M2-polarized TAMs and CAFs also contribute to creating an immunosuppressive environment (32). CAFs contribute by promoting angiogenesis and secreting factors like IL-6 (33).

Nerve remodeling and regeneration: triggers nerve remodeling and regeneration, which paradoxically facilitates further cancer invasion (34). Damage to axons induces the release of factors like neuregulin 1 (NRG1), which activates Schwann cells to proliferate and migrate, creating pathways for tumor cells (8). This process is further stimulated by signals from CAFs, such as SLIT2 and Eph-B, and immune cells, including IL-6, which enhance Schwann cell migration and neuronal plasticity (35). Axon guidance molecules like SEMA3D (20) and the migration of neural precursor cells (36) also contribute to this environment.

**Figure 1:**
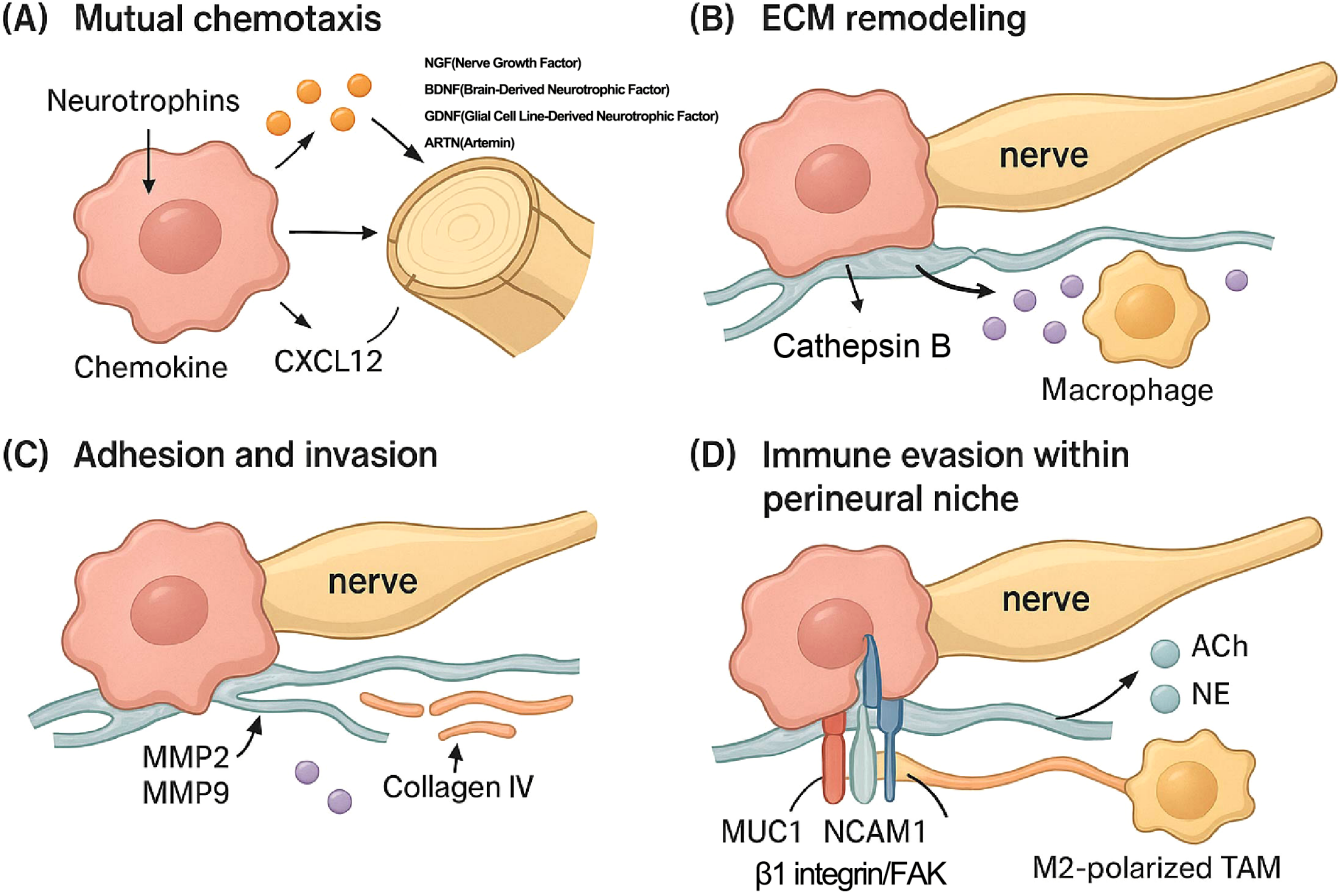
Molecular and cellular mechanisms driving perineural invasion (PNI). This figure details the key molecular and cellular steps of PNI in PDAC. **(A)** Mutual Chemotaxis: Cancer cells secrete neurotrophins (NGF, Nerve Growth Factor; BDNF, Brain-Derived Neurotrophic Factor; GDNF, Glial Cell Line-Derived Neurotrophic Factor; ARTN, Artemin) that promote neurite outgrowth. Conversely, nerves and associated cells release chemoattractants like CXCL12, guiding cancer cell migration toward the nerve. **(B)** ECM Remodeling: Macrophages recruited to the perineural niche secrete proteases, such as Cathepsin B, which degrade extracellular matrix (ECM) components of the nerve sheath (e.g., collagen IV), facilitating cancer cell entry. **(C)** Invasion: Cancer cells secrete matrix metalloproteinases (MMP2, MMP9) to further degrade the ECM and invade the perineural space. **(D)** Adhesion and Immune Evasion: Within the perineural niche, neurotransmitters (ACh, acetylcholine; NE, norepinephrine) released from nerves suppress local immune responses. M2-polarized tumor-associated macrophages (TAMs) further contribute to this immune suppression. Adhesion of the cancer cell to the nerve is critically mediated by molecules expressed on the cancer cell surface, such as Mucin 1 (MUC1), NCAM1, and the β1 integrin/focal adhesion kinase (FAK) signaling complex.

Therefore, PNI is not a passive process but an active, orchestrated invasion driven by complex reciprocal signaling between cancer cells, nerves, and the TME, as illustrated in **Figure 1**. This molecular interplay must be the focus of future therapeutic strategies.

### 2.3 Clinical impact: PNI, pain, recurrence, and prognosis

The clinical consequences of PNI are significant. It is a primary mechanism underlying the severe, often difficult to manage, abdominal and back pain experienced by a majority (up to 80%) of PDAC patients (12). This pain is often neuropathic in nature, resulting from direct nerve damage, inflammation within the perineural niche, and sensitization of nerve endings by mediators released from cancer and immune cells (16). Beyond pain, PNI serves as a critical pathway for tumor dissemination, facilitating local spread and contributing significantly to the high rates of local and regional recurrence observed even after surgery (5). The presence of residual cancer cells within nerve sheaths after resection is thought to be a major factor in treatment failure (37). Consistent with its role in invasion and recurrence, PNI is widely recognized as an independent negative prognostic factor in PDAC (12), correlated with shorter overall survival (OS) and disease-free survival (DFS) (38).

## 3 The immunologically “cold” tumor microenvironment of PDAC

**Figure 2:**
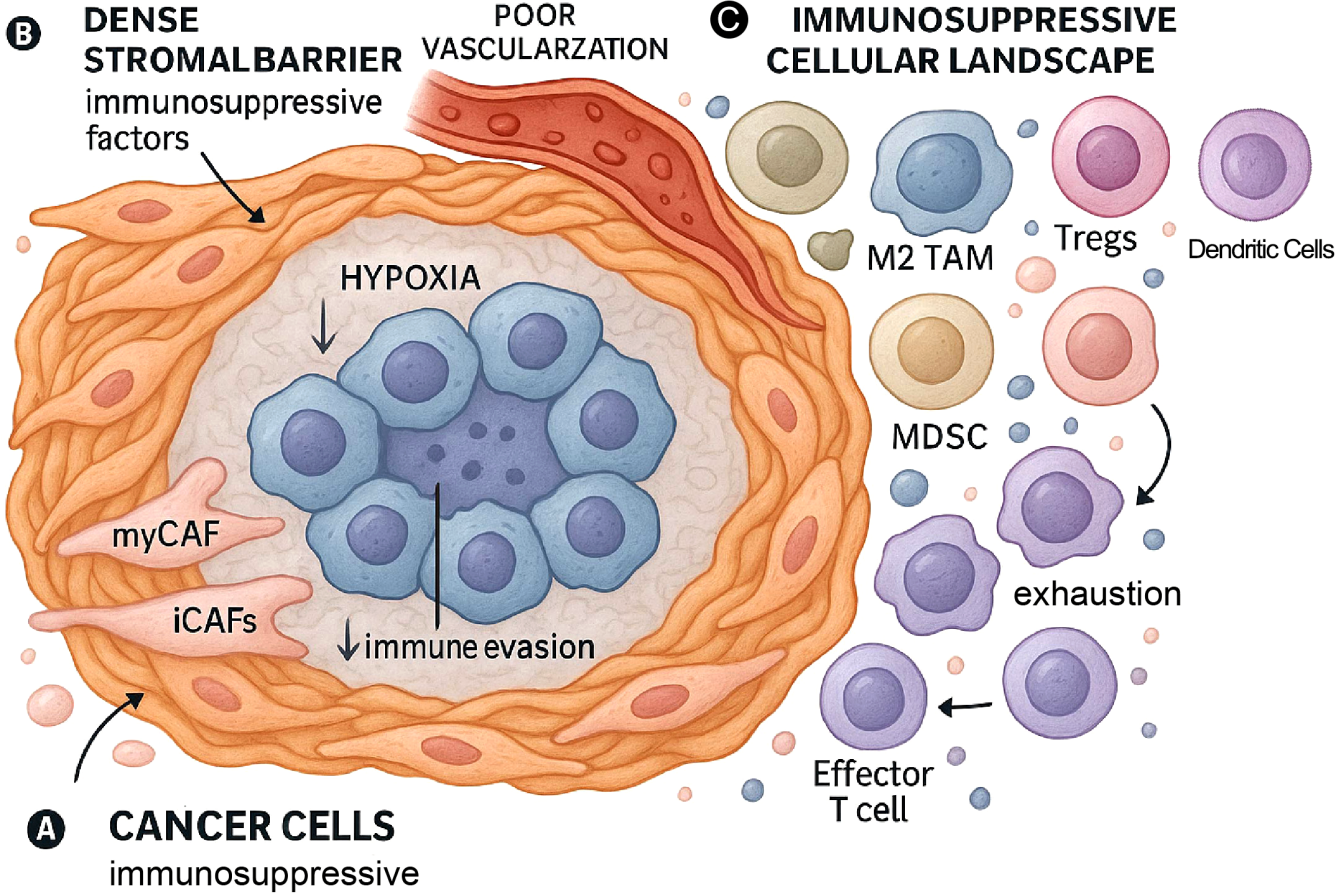
The immunologically “cold” tumor microenvironment (TME) of PDAC. This figure illustrates the key components that establish the immunosuppressive TME in PDAC. **(A)** Cancer Cells: Tumor cells themselves contribute to the immunosuppressive environment through various mechanisms. **(B)** Dense Stromal Barrier & Hypoxia: The TME is characterized by a dense desmoplastic stroma, largely produced by cancer-associated fibroblasts (CAFs). This stroma forms a physical barrier that, along with poor vascularization, leads to hypoxia and impedes immune cell infiltration. **(C)** Immunosuppressive Cellular Landscape: The TME is dominated by immunosuppressive cells, including phenotypically diverse CAFs, M2-polarized TAMs that exist on a spectrum of activation states, myeloid-derived suppressor cells (MDSCs), regulatory T cells (Tregs), and dysfunctional dendritic cells (DCs). This environment is characterized by a scarcity of functional effector T cells.

The TME of PDAC is a complex ecosystem comprising cellular and non-cellular components that profoundly influence tumor biology (1) and establish a profoundly immunosuppressive state (15), as illustrated in **Figure 2**.

### 3.1 Cellular and non-cellular composition: architects of immunosuppression

The immunosuppressive landscape of the PDAC TME is orchestrated by several key components:

Dense stroma (Desmoplasia): PDAC is notorious for its extensive desmoplastic reaction, a dense fibrotic stroma that can account for the vast majority of the tumor volume (14). This stroma is primarily composed of excessive ECM proteins, such as collagen, produced by CAFs (33). The dense matrix creates a physical barrier that hinders the infiltration of effector immune cells (15) and limits the efficacy of therapeutic agents (39).

Hypoxia: The dense stroma and compromised vasculature lead to significant regions of hypoxia (low oxygen) within the TME. Hypoxia activates hypoxia-inducible factor (HIF) signaling pathways (40), which not only drive tumor cell adaptation but also contribute significantly to immunosuppression (41).

Immunosuppressive cellular infiltrate: The cellular landscape is dominated by cells that actively suppress anti-tumor immunity:

Cancer-associated fibroblasts (CAFs): These are abundant stromal cells that act as key orchestrators of the TME (21). Beyond producing the ECM, CAFs actively contribute to immune suppression by secreting factors like CXCL12, which can sequester T cells in the stroma, and Transforming Growth Factor-β (TGF-β), a potent immunosuppressive cytokine (14). CAFs exhibit significant heterogeneity, with subtypes like inflammatory CAFs (iCAFs) (19) and myofibroblastic CAFs (myCAFs) having distinct roles in immune modulation and T-cell exclusion (42). The existence of potentially tumor-restraining CAF subsets further complicates therapeutic targeting (21, 43).

Tumor-associated macrophages (TAMs): TAMs are typically the most abundant immune cells within the PDAC TM (44). The traditional M1 (anti-tumor) vs. M2 (pro-tumor) dichotomy is now considered an oversimplification of their complex biology. Emerging evidence highlights a spectrum of activation states and significant functional heterogeneity within TAM populations (45). For instance, the “M2-like” phenotype encompasses multiple distinct subsets (e.g., M2a, M2b, M2c, M2d), and misinterpreting this diversity can impede the development of effective therapies. In PDAC, TAMs are predominantly polarized toward a pro-tumor, immunosuppressive state. They suppress T-cell activity via IL-10, TGF-β, and PD-L1 expression, and deplete essential amino acids like arginine via arginase-1 (Arg1) (46). The polarization and function of TAMs are dynamically regulated by various signals within the TME, including cytokines, metabolic cues, exosomes, and non-coding RNAs (47, 48).

Myeloid-derived suppressor cells (MDSCs): These are a heterogeneous population of immature myeloid cells that potently inhibit the cytotoxic functions of both T cells (15) and NK cells (42).

Regulatory T cells (Tregs): PDAC tumors are often enriched with CD4+FoxP3+ Tregs (15), which actively suppress the proliferation and effector functions of conventional T cells (49).

Dendritic cells (DCs): As professional antigen-presenting cells (APCs), DCs are essential for priming anti-tumor T-cell responses. However, their function is severely compromised in the PDAC TME. The dense stroma can limit their migration, while immunosuppressive factors from CAFs and TAMs inhibit DC maturation and antigen-presenting capacity. This impaired DC function is a key reason for poor T-cell priming and contributes significantly to the “cold” immune landscape (48–50).

Paucity of effector immune cells: A key feature of the “cold” PDAC TME is the scarcity or dysfunction of anti-tumor effector immune cells, particularly cytotoxic CD8+ T lymphocytes (CTLs) (15). This is attributed to physical exclusion, active immunosuppression, and poor immunogenicity due to a low tumor mutational burden (TMB) (51). The few T cells that do infiltrate often display markers of exhaustion (e.g., high PD-1, TIM-3, LAG-3 expression) (52).

### 3.2 Mechanisms underpinning immune evasion and immunotherapy resistance

The immune evasion of pancreatic ductal adenocarcinoma (PDAC) is driven by a multi-layered suppressive TME (21). This barrier consists of a dense stroma that physically blocks T-cell infiltration (53) and a variety of immunosuppressive cells that actively neutralize immune responses (54). This issue is exacerbated by the tumor’s intrinsically low immunogenicity due to a low mutation burden (51), which promotes T-cell exhaustion (55) and is enhanced by the cancer cells’ own escape tactics (56).

**Figure 3:**
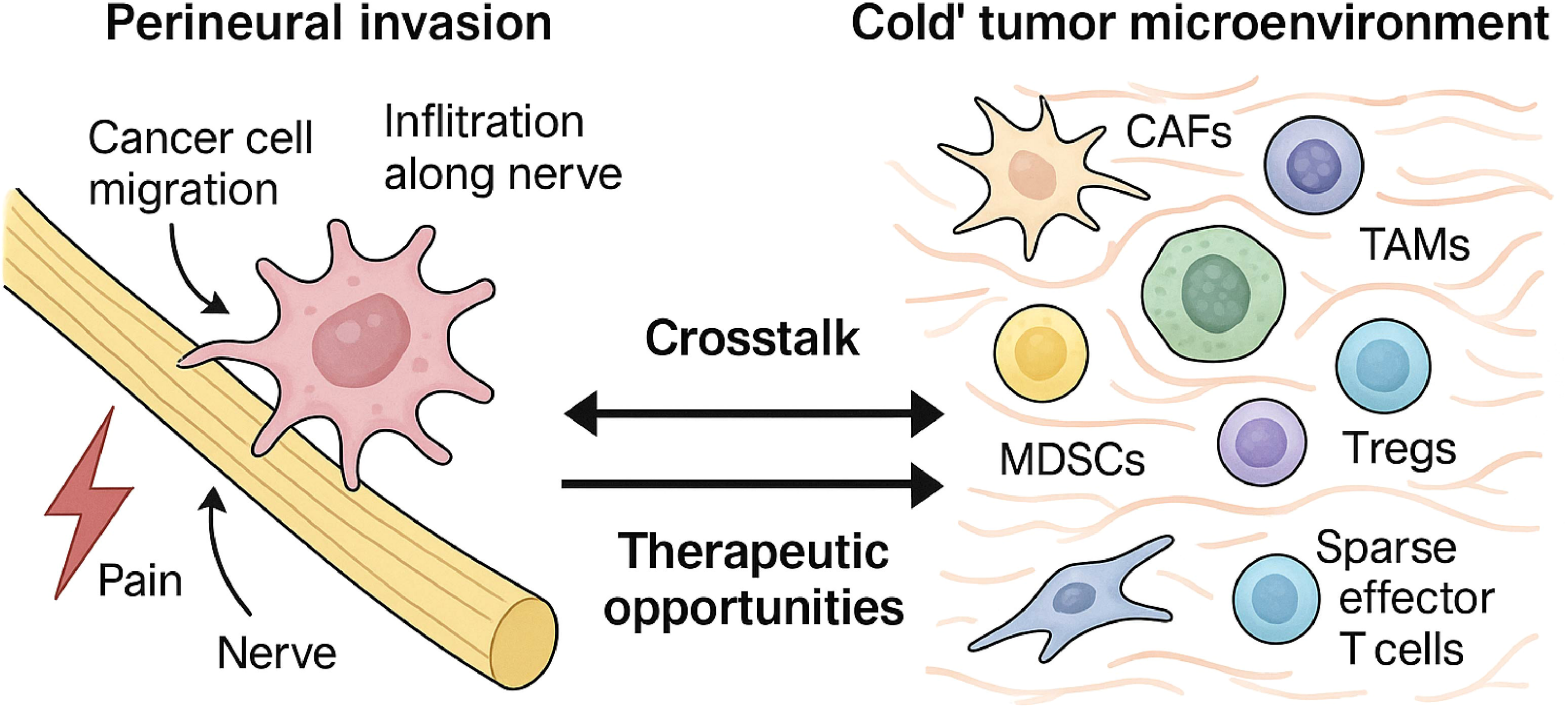
Bidirectional crosstalk between perineural invasion and the immunosuppressive TME. This figure illustrates the profound interconnection between PNI and the TME in PDAC. The left panel depicts PNI, where cancer cells invade and migrate along nerve structures. The right panel illustrates the immunosuppressive TME, populated by CAFs, TAMs, and Tregs, with a notable lack of effector T cells. The central arrows signify the critical bidirectional crosstalk driven by shared molecular mediators—including the CXCL12/CXCR4 axis, TGF-β signaling, and neurotrophin pathways (e.g., NGF/TrkA)—that mechanistically link PNI and immune suppression, creating a self-reinforcing cycle that promotes tumor aggression.

Consequently, PDAC is characterized as an immunologically “cold” tumor, demonstrating profound resistance to ICIs (15). Objective response rates to ICI monotherapy are minimal, typically below 2%, with significant efficacy confined to the rare, more immunogenic subset of tumors with high microsatellite instability (MSI-H/dMMR). The TME is an actively constructed barrier driving multi-therapy resistance (47); therefore, effective treatment strategies must aim to dismantle this suppressive architecture (21), not just stimulate immunity (50). This effort is complicated by substantial TME heterogeneity (56), suggesting that broad targeting may fail and highlighting the need for personalized therapeutic strategies (57). As shown in **Figure 3**, emerging evidence also links this cold TME to perineural invasion through shared molecular and cellular mediators.

## 4 The intricate crosstalk between nerves and the immune microenvironment in PDAC

The realization that PNI and the cold TME are co-conspirators in PDAC progression stems from the growing understanding of the bidirectional communication between neural elements and the immune/stromal components within the tumor (5). This neuro-immune crosstalk involves direct cell interactions and soluble mediators, creating a complex regulatory network.

### 4.1 Neural regulation of the TME: impact of nerves, neurotransmitters, and neurotrophins

Nerves innervating the tumor are not passive structures but actively modulate the TME:

Neurotransmitter modulation of immune cells: Neurons release neurotransmitters that bind to receptors on immune cells, directly influencing their behavior (58).

Norepinephrine (NE): Released by sympathetic nerves, NE generally acts via β-adrenergic receptors to suppress anti-tumor immunity by inhibiting CTL activity (8) and promoting M2 TAM polarization (58).

Acetylcholine (ACh): Released by parasympathetic (vagal) nerves, ACh can impair CD8+ T cell recruitment and function via nicotinic ACh receptors (5).

GABA (Gamma-Aminobutyric Acid): Generally considered inhibitory, GABA can suppress T cell and macrophage activation (58).

Vasoactive Intestinal Peptide (VIP): This neuropeptide can inhibit T cell anti-tumor activity and promote the development of immunosuppressive Treg and Th2 cells (5).

Other Neurotransmitters: Serotonin and dopamine also have complex modulatory effects on T cells and macrophages within the TME (58).

Neurotrophin modulation of immune cells: Neurotrophins, primarily known for their roles in PNI, also interact with the immune system (59):

Nerve growth factor (NGF): NGF and its receptor TrkA are expressed by various immune cells (6). NGF is a key mediator of neurogenic inflammation (60) and can influence T-cell responses (61).

Brain-derived neurotrophic factor (BDNF): BDNF and its receptor TrkB are also implicated in immune modulation, with BDNF being produced by T cells, suggesting autocrine/paracrine loops (62).

Collectively, these neural signals contribute significantly to establishing and maintaining the immunosuppressive TME (17).

### 4.2 TME regulation of neural processes: promoting PNI and neural remodeling

The crosstalk is bidirectional. PDAC cells secrete neurotrophic factors (NGF, BDNF, GDNF) that stimulate neurite outgrowth (8) and attract nerve fibers (63). Stromal and immune cells also contribute significantly:

CAFs and PSCs secrete factors like SLIT2, Tenascin C, and IL-6 that promote neurite outgrowth and Schwann cell migration (8).

TAMs, recruited by Schwann cell-derived CCL2, secrete GDNF, which promotes PNI (5). Mast cells can secrete IL-6, contributing to Schwann cell plasticity (8). Macrophage-derived MIF acting via CD74 can also increase GDNF levels (64). TAMs also release proteases like Cathepsin B that degrade the protective nerve sheath, facilitating invasion (29, 65).

This TME-driven promotion of nerve growth and invasion creates a positive feedback loop that amplifies immunosuppression and stimulates tumor growth (34).

### 4.3 Metabolic interplay in the neuro-immune-cancer axis

Beyond signaling crosstalk, metabolic interactions are emerging as another crucial layer of complexity in the neuro-immune-cancer axis within the PDAC TME (6). Metabolic reprogramming is a fundamental hallmark of cancer, enabling cells to meet the bioenergetic and biosynthetic demands of rapid proliferation in a harsh, nutrient-deprived, and hypoxic environment (66). This reprogramming is heavily influenced by interactions within the TME.

Nerve-cancer metabolic crosstalk: Evidence suggests nerves can directly fuel PDAC progression during PNI. Axons and DRG can secrete serine, an amino acid utilized by PDAC cells for proliferation, particularly under nutrient stress conditions encountered during invasion (8). Glutamate released from nerve endings can activate NMDARs on PDAC cells, triggering downstream signaling (CaMKII/ERK/METTL3) that upregulates hexokinase 2 (HK2), promoting glycolysis (the Warburg effect) and PNI. Furthermore, signaling pathways crucial for PNI, like NGF/TrkA, can directly impact cancer cell metabolism by upregulating glucose transporters like GLUT1, further enhancing glycolytic flux (60).

Immune-cancer metabolic crosstalk: The TME’s metabolic landscape is significantly shaped by immune cells. Immunosuppressive cells like TAMs and MDSCs contribute to the hypoxic and acidic conditions that drive metabolic shifts in cancer cells (66). Moreover, there is intense metabolic competition within the TME. Immunosuppressive cells actively deplete nutrients essential for effector T cell function. For example, MDSCs and TAMs express high levels of Arg1 and iNOS, which consume arginine, an amino acid critical for T cell activation, proliferation, and survival, thereby contributing to T cell dysfunction (44).

Nerve-immune metabolic links: While less explored, potential metabolic interactions between nerves and immune cells within the TME likely exist. Neural signals might influence the metabolic state of immune cells, or vice versa. For instance, metabolic pathways involved in endocannabinoid and polyamine metabolism have been implicated in PNI and associated pain, potentially linking metabolic state to neuro-inflammation (12). The investigation of how nerve-derived metabolites or neurotransmitter signaling impacts immune cell metabolism (e.g., glycolysis vs. oxidative phosphorylation balance in T cells or TAMs) represents an important area for future research.

These findings indicate that the crosstalk governing PDAC progression involves not only complex signaling networks but also intricate metabolic dependencies and competition between cancer cells, nerves, and immune cells. This metabolic interplay likely influences PNI, immune suppression, and overall tumor growth, adding another dimension to the challenge of targeting the TME (20).

The neuro-immune crosstalk in PDAC thus establishes a self-reinforcing cycle driving tumor aggression. Nerves release signals that suppress anti-tumor immunity and promote pro-tumor immune cells, while the TME, including cancer cells, CAFs, and TAMs, secretes factors that stimulate nerve growth and invasion. This increased innervation further amplifies the immunosuppressive signals, creating a vicious loop (5). Breaking this cycle likely necessitates therapeutic strategies that simultaneously target both the neural signaling components and the mechanisms of immune suppression. Furthermore, the specific roles of different nerve types (sympathetic, parasympathetic, sensory) appear complex and context-dependent (17), suggesting that neuro-modulatory therapies must be carefully tailored based on the specific pathways involved and the stage of the disease.

## 5 Shared mediators and pathways orchestrating PNI and immune suppression

The crosstalk between PNI and the cold TME is orchestrated by specific molecular pathways and cellular mediators that function at the interface of these two processes.

### 5.1 The CXCL12/CXCR4 axis

The CXCL12/CXCR4 chemokine axis is pivotal in PDAC pathogenesis, driving both perineural invasion (PNI) and immune evasion (21). In the tumor microenvironment, CXCL12 secreted by stromal cells like CAFs/PSCs and potentially nerves (8) attracts CXCR4-overexpressing PDAC cells, guiding their migration toward neural structures and facilitating PNI (21). This interaction is clinically significant, as high CXCL12/CXCR4 expression is an independent negative prognostic factor associated with PNI (38), while the atypical receptor ACKR3 also promotes invasion (67). Concurrently, this axis creates an “immune-excluded” phenotype by using stromal CXCL12 to sequester CXCR4-positive T cells, preventing their infiltration into tumor nests (68). The axis’s immunomodulatory role is complex, as high CXCR4 expression, despite influencing the trafficking of immune cells like macrophages (66), also correlates with elevated inhibitory checkpoints such as PD-1/PD-L1, fostering a suppressed immune state (38). Thus, the CXCL12/CXCR4 axis functions as a critical node linking stromal activation with cancer cell invasion and profound immune dysfunction.

### 5.2 Transforming growth factor-β signaling

TGF-β is a pleiotropic cytokine that critically promotes both PNI and immune suppression within the PDAC TME (47). For PNI, TGF-β from stromal sources like Schwann cells and CAFs directly enhances PDAC cell invasive capacity (8), while also driving desmoplastic ECM remodeling and inducing an EMT program that increases cell motility (56). Simultaneously, TGF-β is one of the most potent immunosuppressive cytokines in the TME, secreted by cancer and stromal cells to inhibit the function of cytotoxic T cells and NK cells (56), promote regulatory T cells (Tregs), and polarize macrophages toward a suppressive M2 phenotype (69). The complexity of its role is highlighted by the “TGF-β paradox,” where its function switches from tumor-suppressive to pro-oncogenic during disease progression (56), a process influenced by factors like the frequent loss of SMAD4 in PDAC (70). Furthermore, non-canonical signaling pathways can promote aggressive phenotypes and PD-L1 upregulation (71). Thus, TGF-β signaling serves as a central node, fostering key mechanisms of PNI while orchestrating a profoundly immunosuppressive microenvironment.

### 5.3 Neurotrophin signaling pathways (NGF/TrkA, BDNF/TrkB)

These pathways are fundamental for the mutual chemotaxis that initiates PNI (8). Activation of Trk signaling promotes cancer cell proliferation, migration, and invasion (72). The NGF/TrkA pathway is also a key mediator of PNI-associated pain (12). These neurotrophins and their receptors are also expressed on various immune cells (59), participating in the complex neuro-immune dialogue (73).

### 5.4 Cancer-associated fibroblasts: heterogeneity and dual roles

CAFs are central, pleiotropic cells in the PDAC TME, actively driving both PNI and immune suppression (74). They facilitate PNI by producing and remodeling the dense ECM to create invasive tracks (8), with subtypes like αSMA-high myCAFs providing direct physical support (75), and by secreting numerous pro-invasive and neurotropic factors, including TGF-β, HGF, and SLIT2. As major architects of immunosuppression (76), CAFs construct a physical barrier, release potent inhibitory cytokines like TGF-β and IL-6 (77), recruit cells such as MDSCs and Tregs, and directly impair T cells via mechanisms like CXCL12 secretion (68). This dual functionality is governed by significant CAF heterogeneity: iCAFs are linked to inflammation and immunosuppression, myCAFs primarily contribute to ECM deposition, and apCAFs may induce T cell tolerance (75), with the existence of tumor-restraining subtypes further underscoring the complexity (78). Thus, CAFs represent a critical cellular hub that physically and chemically engineers the TME to promote invasion while orchestrating profound immunosuppression.

### 5.5 Tumor-associated macrophages: heterogeneity and dual roles

TAMs are abundant and functionally diverse cells that bridge PNI and immune suppression, often adopting an M2-like phenotype upon recruitment by factors such as CCL2 or CSF-1 (44). They actively facilitate PNI by degrading nerve barriers with enzymes like Cathepsin B, releasing pro-invasive factors like GDNF (8), and interacting with CAFs through mechanisms like LIF signaling to contribute to neural remodeling (79). Concurrently, M2-polarized TAMs are cornerstone immunosuppressive cells in the PDAC TME, potently inhibiting T and NK cell activity through secretion of IL-10 and TGF-β, expression of PD-L1, and recruitment of Tregs via chemokines like CCL22 (80). This convergence of PNI and immune suppression is driven by such shared cellular mediators and signaling nodes, with pleiotropic cells like TAMs and CAFs and pathways like CXCL12/CXCR4 and TGF-β mechanistically linking both processes (21). Therefore, targeting these central players offers a compelling ‘double hit’ strategy to simultaneously disrupt PNI and alleviate TME immunosuppression (**Table 1**).

**Table 1:** Key molecular pathways and cellular mediators linking PNI and immune suppression in PDAC.

Pathway/Mediator	Role in PNI	Role in immune suppression	Key references
CXCL12/CXCR4 Axis	- Promotes cancer cell chemotaxis toward nerves (21) - Facilitates invasion & potentially EMT (21) - High expression correlates with PNI & poor prognosis (38)	- Sequesters/traps T cells in the stroma, preventing tumor infiltration (68) - Influences immune cell trafficking (T cells, MΦ) (81) - Associated with high immune checkpoint expression (38)	(21)
TGF-β Signaling	- Enhances cancer cell invasion (8) - Drives ECM production/remodeling by CAFs (8) - Induces EMT (56)	- Potent inhibitor of CTL & NK cell function (82) - Promotes Treg development & function (82) - Drives M2 TAM polarization (82) - Contributes to fibrosis & immune evasion (83) - Non-SMAD signaling upregulates PD-L1 (71)	(8)
NGF/TrkA Signaling	- Mediates mutual chemotaxis (8) - Promotes cancer cell proliferation, migration, invasion, and survival (72) - Major mediator of PNI-associated pain (16) - Overexpression correlates with PNI & poor prognosis (72)	- Modulates neurogenic inflammation (73) - TrkA expressed on activated T cells, potential role in T cell function (74) - Activates mast cells (74)	(8)
CAFs (Heterogeneous)	- Major ECM producers/remodelers (collagen, MMPs) (8) - Secrete factors promoting chemotaxis (Tenascin C) & invasion (TGF-β, HGF) (8) - Drive nerve remodeling (SLIT2, IL-6, Eph-B) (8) - myCAFs often adjacent to invading cells (75)	- Create physical barrier (dense stroma) (75) - Secrete immunosuppressive factors (TGF-β, IL-6) (77) - Recruit MDSCs, Tregs (68) - Exclude/inhibit T cells (via CXCL12, FAP) (68) - Subtype-specific roles (iCAF vs myCAF vs apCAF) (42)	(8)
TAMs (Mainly M2-like)	- Degrade nerve sheath (Cathepsin B) (5) - Promote cancer cell invasion/migration (GDNF via RET/ERK) (5) - Recruited by Schwann cells (CCL2) (8) - Contribute to neural remodeling (via LIF) (5)	- Suppress T/NK cell activity through a variety of mechanisms reflecting their plasticity (e.g., IL-10, TGF-β, Arg1, PD-L1) (68) - Recruit Tregs (via CCL22) (80) - Promote angiogenesis & metastasis (44) - Contribute to chemoresistance (84)	(5)

MΦ, Macrophage.

## 6 Therapeutic strategies targeting the PNI-immune TME axis

**Figure 4:**
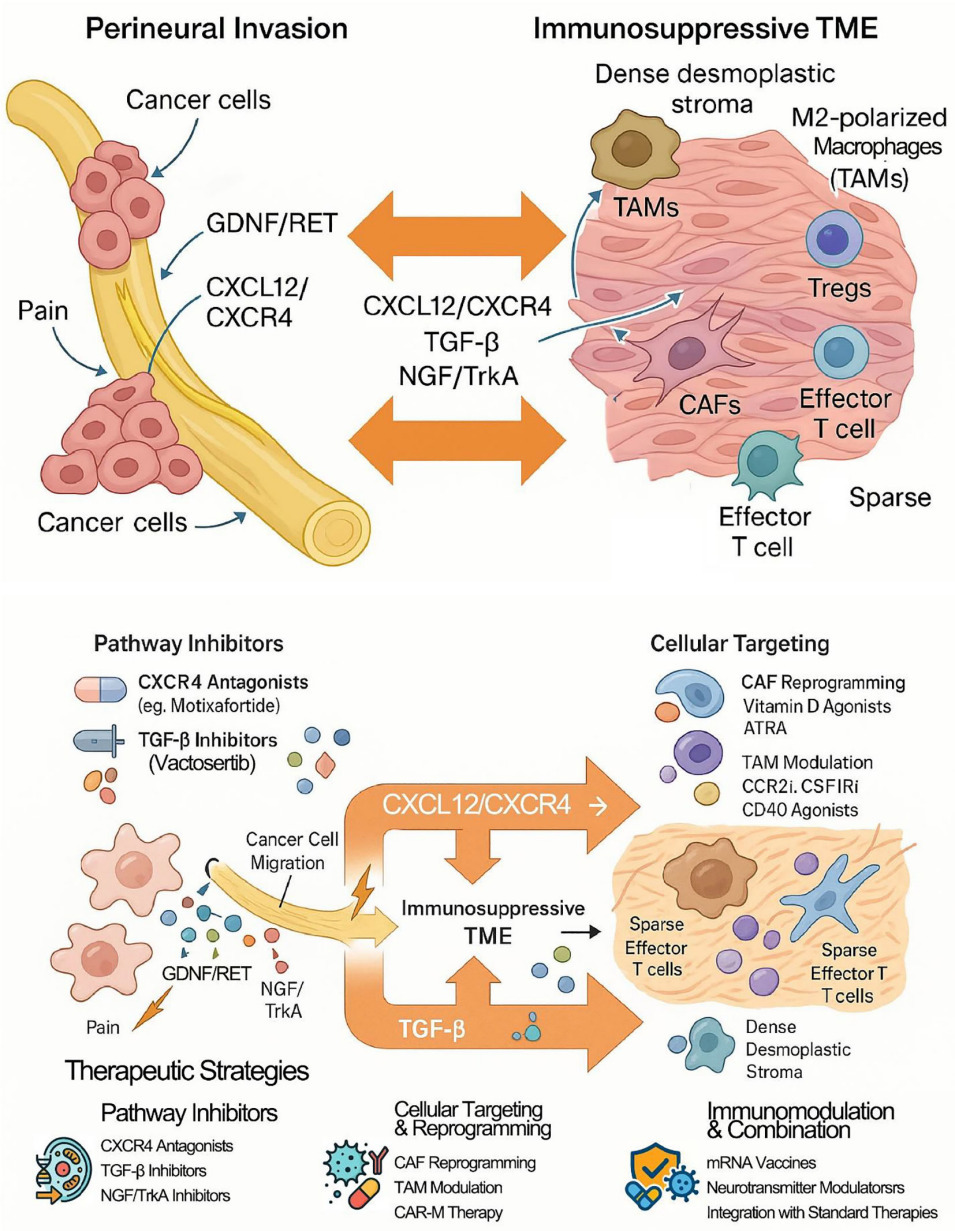
Neuro-immune crosstalk and therapeutic targets in pancreatic cancer. In pancreatic cancer (PDAC), perineural invasion (PNI) and the immunosuppressive tumor microenvironment (TME) form a vicious cycle driven by crosstalk through shared cells (e.g., CAFs, TAMs) and signaling pathways (e.g., CXCL12/CXCR4, TGF-β) that promotes tumor progression. Therapeutic strategies aim to disrupt this cycle by inhibiting key pathways, reprogramming stromal cells, and integrating emerging immunotherapies like mRNA vaccines. The ultimate goal is to simultaneously inhibit PNI while converting the immunologically "cold" TME into a "hot," therapy-responsive state.

Targeting the shared mediators of the PNI-TME crosstalk offers a promising strategy to simultaneously disrupt tumor invasion and “heat up” the cold TME (21). As illustrated in **Figure 4**, targeting the shared molecular pathways and cellular mediators that orchestrate the PNI-TME crosstalk offers promising strategies to simultaneously disrupt tumor invasion and ‘heat up’ the cold TME.

### 6.1 Targeting shared molecular pathways

CXCR4 antagonists: Blocking the CXCL12/CXCR4 axis with agents like Plerixafor or Motixafortide aims to inhibit PNI and enhance T cell infiltration (85). The Phase IIa COMBAT trial (NCT02826486), combining Motixafortide with pembrolizumab and chemotherapy, showed promising signals of activity in metastatic PDAC (86).

TGF-β inhibitors: Inhibiting TGF-β signaling is an attractive strategy to reduce fibrosis and alleviate immunosuppression (70). However, the clinical development of some agents has faced challenges. For example, a Phase Ib trial combining the TGFBR1 inhibitor galunisertib with durvalumab showed limited clinical activity (87). The sentence structure describing the trial outcome has been corrected for clarity: The combination was tolerable, but clinical activity was limited (1 partial response, 7 stable diseases out of 32 patients), with a median PFS of 1.87 months and mOS of 5.7 months (87). It is noteworthy that the development of galunisertib for oncology indications was later discontinued. Other TGF-β inhibitors, such as the small molecule inhibitor Vactosertib or monoclonal antibodies targeting TGF-β, are under investigation in various cancers, representing alternative approaches to target this pathway (88).

Trk inhibitors: Targeting neurotrophin signaling, particularly NGF/TrkA, is under preclinical investigation as a strategy to inhibit PNI and alleviate associated pain (59). In mouse models, combining Trk inhibition with gemcitabine increased survival (89). Pan-Trk inhibitors like Larotrectinib are approved for rare TRK fusion-positive cancers, but preclinical studies using specific TrkA inhibitors or NGF-neutralizing antibodies have shown they can reduce the PNI potential of PDAC cells and inhibit neurite outgrowth (72).

### 6.2 Modulating key cellular players

Targeting CAFs: Given their heterogeneity, strategies are shifting from broad depletion to selective targeting or reprogramming (75). Approaches include targeting Fibroblast Activation Protein (FAP) or reprogramming CAFs toward a quiescent state using agents like Vitamin D receptor agonists (e.g., Calcipotriol) or all-trans retinoic acid (ATRA) (90) (91). Clinical trials investigating these approaches are ongoing (e.g., NCT03520790). More refined approaches aim to deplete subsets like Pdgfrb+ CAFs or adipose marker-expressing (ASC-like) CAFs, with the latter showing potential to enhance ICI efficacy in preclinical models (42). Targeting key signaling pathways within CAFs (e.g., JAK/STAT, HGF/c-Met) or inhibiting their ECM-modifying functions (e.g., LOX inhibitors) are also under investigation (92).

Targeting TAMs: Modulating the abundant and largely immunosuppressive TAM population is a key strategy to reprogram the TME (44). Approaches include inhibiting monocyte recruitment by blocking receptors like CCR2 and CSF1R (84), with a clinical trial combining the CCR2 antagonist PF-04136309 with FOLFIRINOX showing encouraging results in locally advanced PDAC (93); depleting existing TAMs via agents targeting CSF1R or other macrophage-specific markers (84); reprogramming M2 to M1 phenotypes using agents like TLR or CD40 agonists (94); and enhancing phagocytosis by blocking “don’t eat me” signals such as the CD47-SIRPα interaction (95). Targeting TAMs is frequently explored in combination with ICIs or other immunotherapies, aiming to reduce a major source of immunosuppression within the TME (44). Additionally, novel strategies such as CAR-macrophage (CAR-M) therapy are emerging, which engineer macrophages to directly target and phagocytose tumor cells (96, 97).

### 6.3 Neurotransmitter modulation strategies

Targeting neurotransmitter signaling is an emerging avenue:

β-blockers: Antagonizing NE signaling with drugs like propranolol has shown preclinical potential to reduce PNI and increase survival when combined with chemotherapy in KPC mouse models (a genetically engineered mouse model expressing oncogenic Kras and mutant Trp53, specifically KrasLSL-G12D/+; Trp53LSL-R172H/+; Pdx1-Cre) (89) (58). However, clinical data on the impact of β-blockers on cancer outcomes have been inconsistent, highlighting the need for a better understanding of specific β-AR subtype roles and patient selection (62).

VIP antagonists: Preclinical studies showed that blocking VIP signaling could synergize with anti-PD-1 therapy, enhancing T cell activation and recruitment (5).

### 6.4 Emerging strategies: mRNA neoantigen vaccines

A significant frontier in overcoming the poor immunogenicity of PDAC is personalized cancer vaccination. Recent breakthroughs with mRNA vaccine technology have shown remarkable promise. A notable study demonstrated that a personalized mRNA neoantigen vaccine (autogene cevumeran) could induce a substantial population of durable, polyfunctional CD8+ T cells targeting tumor-specific neoantigens in PDAC patients (98, 99). These vaccine-induced T cells persisted for up to two years and were associated with delayed tumor recurrence. This approach directly addresses the lack of pre-existing T-cell responses that limit ICI efficacy. By generating a potent *de novo* T-cell response, mRNA vaccines could potentially “heat up” the cold TME, making it more susceptible to checkpoint inhibition and other immunotherapies. Integrating such vaccination strategies with therapies that target the stromal and neural barriers of the TME represents a powerful future direction for combination treatments.

### 6.5 Combination therapies: disrupting PNI and “heating” the TME

Due to the complex nature of pancreatic cancer resistance, driven by PNI, stromal barriers, and immunosuppression, combination therapies are crucial for clinical advancement (100). Key strategies involve pairing agents that disrupt nerve-cancer signaling pathways, such as inhibitors of CXCR4, TGF-β, or Trk, with immunotherapies to amplify T cell responses (89). Another approach combines agents that modulate stromal cells like CAFs or TAMs with immune checkpoint inhibitors (ICIs) to dismantle physical and cellular barriers to immunity (44). Preclinical studies have also demonstrated durable responses by combining CXCR1/2 inhibition with T cell activating agents, or by targeting neurotransmitter pathways alongside ICIs (101).

These targeted combinations are frequently integrated with standard chemotherapy or radiotherapy. This integration aims to leverage the immunogenic cell death induced by conventional treatments, which can release tumor antigens and synergize with immunotherapy, despite the potential for inducing resistance (68). Examples include adding CXCR4 or CCR2 inhibitors to standard chemotherapy regimens, with or without ICIs (86) (93). The ultimate goal of these multifaceted strategies is to transform the immunologically “cold” and resistant tumor microenvironment into an inflamed, “hot” state that is susceptible to immune-mediated destruction, while simultaneously inhibiting PNI to control local invasion and recurrence (21).

### 6.6 Preclinical and clinical evidence landscape

While preclinical studies have generated considerable enthusiasm, translation into significant clinical benefit has remained challenging (102). Key clinical trials investigating these strategies include:

CXCR4 inhibition: The COMBAT trial (NCT02826486) showed promising signals of activity for Motixafortide combined with immunotherapy and chemotherapy (86).

TGF-β inhibition: The trial of Galunisertib plus Durvalumab (NCT02734160) was tolerable but had limited efficacy in pre-treated metastatic patients, and the drug’s development was not pursued for this indication (87).

CCR2 inhibition: Combining the CCR2 antagonist PF-04136309 with FOLFIRINOX (NCT01413022) showed potential benefit in locally advanced PDAC (93).

CAF/stroma targeting: Trials involving hyaluronidase inhibitors (PEGPH20) failed to show benefit in Phase III, potentially due to a lack of patient selection based on hyaluronan levels (97). Trials with Vitamin D analogues are ongoing (103).

ICI combinations: Numerous trials combining ICIs with other agents have yielded modest results overall, except for the rare subset of MSI-H PDAC (100). An ongoing Phase III trial (JCOG1908E) is assessing chemo-radiotherapy with or without durvalumab in locally advanced PDAC (104).

Major hurdles impeding clinical success include the profound heterogeneity of the PDAC TME, the lack of validated predictive biomarkers, difficulties in achieving adequate drug delivery, and determining optimal combination strategies (100). Strategies focusing on reprogramming rather than simple elimination, guided by precise biomarkers reflecting the TME state, may be more successful (**Table 2**).

**Table 2:** Selected therapeutic strategies targeting the PNI-immune TME axis in PDAC.

Therapeutic target/strategy	Example agent(s)	Mechanism (PNI/immune modulation)	Development stage	Key references/trials
CXCR4	Motixafortide (BL-8040), Plerixafor	- Inhibit PNI (cancer cell chemotaxis) - Disrupt T cell sequestration, enhance infiltration	Phase II (Combination)	(21)
TGF-β Signaling	Vactosertib, anti-TGFβ mAbs (Galunisertib development discontinued)	- Inhibit invasion/EMT? Reduce fibrosis - Alleviate immunosuppression (Treg↓, CTL/NK↑)	Phase Ib/II (Combination)	(82)
TrkA (NGF Receptor)	GW441756 (preclin), Larotrectinib	- Inhibit PNI (chemotaxis, migration) - Reduce PNI-associated pain - Modulate neurogenic inflammation?	Preclinical (for PDAC PNI)	(72)
CAFs (FAP+)	FAP-CAR-T, Sibrotuzumab	- Deplete specific CAF subset - Reduce ECM, immunosuppression?	Preclinical/Phase I/II	(91)
CAFs (Reprogramming)	Calcipotriol, ATRA	- Revert CAFs to quiescent state - Reduce fibrosis, immunosuppression	Preclinical/Phase I/II	(90)(NCT03520790)
CCR2 (Monocyte/TAM Rec.)	PF-04136309	- Inhibit M2 TAM recruitment - Reduce immunosuppression	Phase Ib/II (Combination)	(84)(NCT01413022)
CSF1R (TAM Survival/Diff.)	Pexidartinib, Emactuzumab	- Deplete/reprogram TAMs - Reduce immunosuppression	Phase I/II (Combination)	(44)
CD40 (APC/TAM Activation)	Sotigalimab (APX005M)	- Activate APCs (DCs) - Reprogram TAMs to M1?	Phase Ib/II (Combination)	(94)
CAR-Macrophages (CAR-M)	N/A (platform)	- Engineered phagocytosis of tumor cells - TME remodeling	Preclinical/Phase I	(96, 97)
Neoantigen Vaccination	Autogene cevumeran (mRNA vaccine)	- Prime *de novo* tumor-specific CD8+ T cell responses	Phase I/II	(98, 99)
VIP Receptor (VIP-R)	VIP-R Antagonist Peptides	- Block T cell inhibition by VIP - Enhance T cell activation/recruitment, reduce exhaustion	Preclinical	(5)
β-Adrenergic Receptors	Propranolol (non-selective)	- Inhibit NE signaling - Reduce tumor growth/PNI? Reduce immunosuppression?	Preclinical/Observational	(58)
Combination Example 1	CXCR4i + Chemo + anti-PD1	- Block PNI, enhance T cell access - Combine with chemo + ICI	Phase II	(86)(COMBAT Trial)
Combination Example 2	CCR2i + FOLFIRINOX	- Reduce TAM recruitment - Combine with standard chemotherapy	Phase Ib/II	(93)(NCT01413022)
Combination Example 3	VIP-R Antag. + anti-PD1	- Block neural T cell inhibition - Enhance ICI efficacy	Preclinical	(5)

ATRA, All-trans retinoic acid; APC, Antigen-presenting cell; CAR-T, Chimeric antigen receptor T cell; CTL, Cytotoxic T lymphocyte; DC, Dendritic cell; ICI, Immune checkpoint inhibitor; NK, Natural killer cell; MΦ, Macrophage.

## 7 Conclusion and future perspectives

### 7.1 Synthesizing the central role of PNI-TME crosstalk

The evidence reviewed herein strongly supports the conclusion that PNI and the immunologically cold TME are not independent pathological features of PDAC, but are deeply interwoven through extensive and bidirectional crosstalk (20). This neuro-immune axis is fundamental to PDAC’s aggressive biology. Shared signaling pathways and pleiotropic cellular players, particularly CAFs and the functionally diverse TAMs, act concertedly to orchestrate PNI while establishing profound immunosuppression (21). This interplay drives invasion, metastasis, therapy resistance, and critically underlies the failure of immunotherapies in most patients (12).

### 7.2 Key challenges and unanswered questions

Despite significant progress, substantial challenges remain:

Heterogeneity: The remarkable heterogeneity of PDAC—particularly within CAF and TAM populations—poses a major obstacle (75). Understanding how this impacts PNI-TME crosstalk and therapy response is crucial.

Biomarkers: There is an urgent need for validated biomarkers that can accurately reflect the state of the entire neuro-immune axis, not just cancer cell-centric markers, to guide patient stratification for TME-targeted therapies (2).

Mechanism nuances: A deeper understanding of the context-dependent roles of different nerve types, neurotransmitters, and specific CAF/TAM subsets is required (17). The contribution of metabolic crosstalk also warrants further investigation (6).

Therapeutic translation: Significant hurdles remain in translating promising preclinical findings into effective clinical therapies. These include optimizing drug delivery through the dense stroma, designing rational and tolerable combination regimens with appropriate sequencing, managing potential toxicities associated with targeting pathways with physiological roles, and improving clinical trial design to account for TME heterogeneity (39).

### 7.3 Future research directions and therapeutic outlook

Addressing these challenges requires a multi-pronged approach focused on deeper mechanistic understanding and smarter therapeutic design:

Advanced modeling and analysis: Continued use of sophisticated preclinical models combined with cutting-edge analytical tools (e.g., single-cell multi-omics, spatial transcriptomics) is essential to dissect PNI-TME interactions (8).

Precision targeting: Future therapies should move toward selectively targeting or reprogramming specific detrimental cell subsets (e.g., iCAFs, immunosuppressive TAMs), guided by robust biomarkers (76).

Rational combination therapies: The focus must be on designing mechanism-based, synergistic combinations. This includes strategically combining agents that target different nodes of the PNI-TME axis—for instance, pairing stromal modulators with ICIs, or integrating novel approaches like personalized mRNA vaccines to generate T-cell responses and CAR-M therapy to directly engage tumor cells and remodel the microenvironment (98).

Early intervention: Investigating the efficacy of targeting this axis in neoadjuvant or adjuvant settings may offer a crucial window of opportunity to prevent disease progression and recurrence.

In conclusion, while PDAC remains a formidable clinical challenge, the growing appreciation of the critical crosstalk between perineural invasion and the immunosuppressive TME offers new avenues for therapy. By continuing to unravel the complexities of this neuro-immune axis and developing rational therapeutic strategies to disrupt its detrimental effects, there is significant potential to improve the prognosis for patients with this disease (21).
